# Leaf trait plasticity and site-specific environmental variability modulate the severity of visible foliar ozone symptoms in *Viburnum lantana*

**DOI:** 10.1371/journal.pone.0270520

**Published:** 2022-07-26

**Authors:** Michele Faralli, Fabiana Cristofolini, Antonella Cristofori, Marco Ferretti, Elena Gottardini

**Affiliations:** 1 Center Agriculture Food Environment (C3A), University of Trento, San Michele all’Adige, Italy; 2 Department of Biodiversity and Molecular Ecology, Research and Innovation Centre, Fondazione Edmund Mach (FEM), San Michele all’Adige, Trento, Italy; 3 Swiss Federal Institute for Forest Snow and Landscape Research, Birmensdorf, ZH, Switzerland; Ilam University, ISLAMIC REPUBLIC OF IRAN

## Abstract

The assessment of Visible Foliar Symptoms (VFS) is commonly adopted by forest monitoring programs to evaluate ozone impact on vegetation. The occurrence of ozone VFS may differ among individuals of the same species at the same site, and within leaves of the same individual. The aim of this study was to identify site and plant characteristics as well as functional leaf traits associated with the occurrence and severity of VFS in *Viburnum lantana* (an ozone-sensitive species) and at the scale of an individual site. *V*. *lantana* plants growing at one site of the ViburNeT monitoring network (Trentino, North Italy) experiencing high ozone levels were surveyed in relation to 1) sun exposure, 2) shading effect from neighbor vegetation, 3) plant height and 4) presence and severity of VFS. Leaves from three different sections of each plant were subjected to a phenotypic characterization of leaf area, dry weight, specific leaf area (SLA), chlorophyll content (Chl_SPAD_), percentage of VFS, and adaxial and abaxial trichome density (Tr). We showed that plants at high irradiation levels had significantly lower SLA (p<0.05), higher Tr (p<0.01) and greater Chl_SPAD_ (p<0.01) when compared to shaded and/or west- and north-exposed plants, thus indicating a strong influence of site-specific characteristics on leaf trait plasticity. Similar differences were observed for taller vs. shorter plants and apical vs. basal branches (p<0.05). Ozone-induced VFS at leaf level were associated with lower SLA (p<0.001) and higher Tr in the abaxial leaf surface (p<0.05). Both leaf traits showed significant differences also within the south and east exposed plant category, thus suggesting the increase in leaf thickness and Tr as a potential adaptive strategy under multiple stress conditions. Our results provide evidence of a strong relationship between VFS, leaf traits and site-specific variables, offering new insights for interpreting data on the impact of ozone on vegetation.

## 1. Introduction

The assessment of Visible Foliar Symptoms (VFS) attributable to tropospheric ozone (O_3_) is a common approach adopted by forest monitoring programs to evaluate the potential risk and impact caused by ozone on vegetation (e.g. ICP Forests http://icp-forests.net/, US Forest Health Monitoring https://www.fs.fed.us/foresthealth/protecting-forest/forest-health-monitoring/ [cited 2021 December 2]). The ozone-induced VFS corresponds to the collapse and death of groups of cells in the palisade tissue [1]. This process is triggered by the formation of reactive oxygen species (ROS) from ozone degradation after it enters into the mesophyll through the stomata [2]. On angiosperms species, they typically consist of reddish to brownish stippling on the adaxial side of leaf, between the second-order veins, generally more pronounced on older leaves and in the lower position of the branch [1, 3]. Standardized procedures recommend evaluating these general features for the ozone injury diagnosis considering fully developed, light-exposed leaves [4], with the purpose to obtain reliable and comparable data in space and time.

A large variability in ozone sensitivity among woody species has been often observed during field assessment of VFS, (e.g., [5]). Feng et al. [6] partially explain this variability with the interspecific variation in leaf mass per area (LMA = 1/specific leaf area, SLA), since species with high LMA are supposed to be less sensitive to ozone following a dilution effect, i.e. lower ozone load per unit leaf mass [7]. Other functional leaf traits such as the non-glandular trichomes play a protective role against oxidative stresses [8]. Trichome morphology and density in fact may largely vary among plant species and populations, and within individual plants [9, 10] as environmental cues can significantly modulate trichome density [10]. Indeed, they seem to play a role in the ozone sink before it enters the leaf, due to i) the increased active leaf surface and ii) the maintenance of a thick and moist boundary layer [8]. However, outcomes in this respect are contradictory and other authors (e.g. [11]) report that only the density of glandular trichomes is positively correlated with leaf ozone uptake reduction, while non-glandular trichomes do not show any protective role against ozone. Part of this inconsistency may be attributed to the controlled environmentally nature of most studies focusing on understanding the putative protective role of trichomes under developing stress conditions, and thus extensive field studies may help in understanding their functions.

It is commonly reported that the occurrence of VFS can differ also within the same species at the same site, and within leaves of the same plant [12]. Yet, very few attempts, if any, exist to investigate which factors are associated with the occurrence of VFS on different plants of the same species growing at the same sites, where site condition and ozone exposure levels are supposed to be similar. Variability of VFS at site level has been reported for *Viburnum lantana* [13], a native species sensitive to ozone, and for which VFS have been experimentally validated and documented over the past decades [3, 14–17]. Previous in-field studies showed that the frequency of ozone VFS on native *V*. *lantana* plants is consistent with the ozone exposure levels within the same growing season [13], over distinct growing seasons [18] and even at the meso-scale spatial resolution [19].

Here we focus on the identification of factors associated with the occurrence and severity of VFS at the scale of individual site. For this study, we selected one of the n = 5 sites of the *V*. *lantana* ozone biological response Network in Trentino (Italy) (*ViburNeT*) [18, 19] with the highest AOT40 (i.e. above 9,000 ppb h) estimated values. The site was characterized by an average of 32.5% of symptomatic *V*. *lantana* plants over the eight field assessment campaigns carried out since 2010 (min 10%; max 83%).

We investigated whether: (i) site-specific conditions (shading, aspect–see below) and plant-related (size, stem portion) variables affect *V*. *lantana* leaf traits; (ii) the presence and intensity of VFS on *V*. *lantana* leaves (i.e. stipple) are associated with morphological and physiological (functional) leaf traits (SLA, trichomes, chlorophyll content); (iii) site-specific (shading, aspect) and plant-related (size, stem portion) variables influence the occurrence of VFS on *V*. *lantana*.

Although carried out locally, results can have a much broader relevance for planning and interpreting field studies, and for improving sampling design in terms of plant and leaf selection for the VFS assessment.

## 2. Materials and methods

### 2.1 Study site

**Fig 1:**
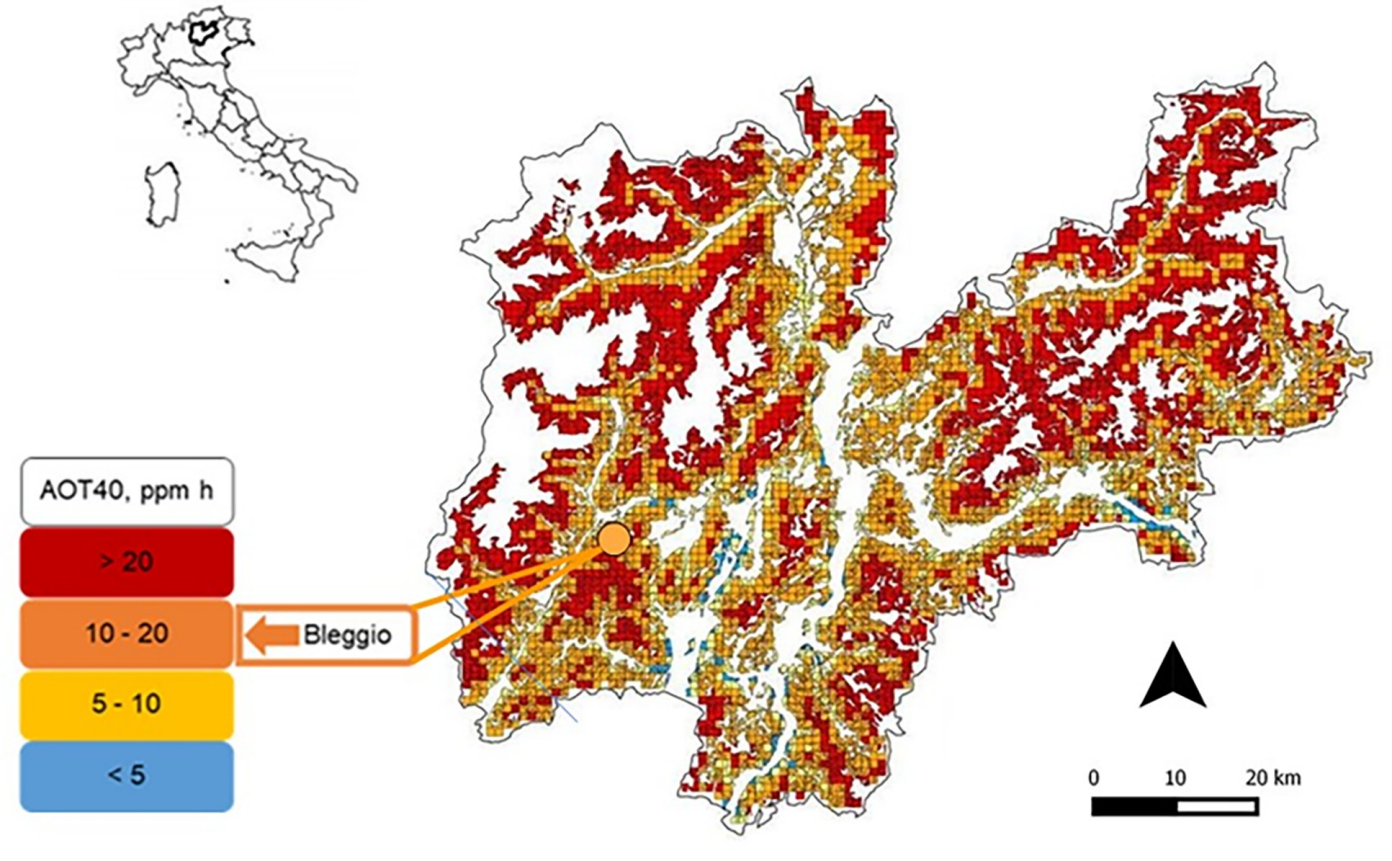
Study site. Location of Trento province in Italy (top left), and of the Bleggio study site within Trento province (right). Ozone was estimated for forest areas with a 1x1 km square resolution, on the basis of a modeling exercise based on a four-year measurement campaign (2007–2010) [18]. Trento province map is colored based on four AOT40 levels (see legend), and the estimate for the Bleggio site is shown on the bottom left.

The study was performed in the Trento province (North Italy) at Bleggio (~2 ha), one of the n = 5 sites included in the *ViburNeT* network with the highest AOT40 (i.e. above 9,000 ppb h) estimated values, implemented in 2010 and yearly assessed for the presence of ozone-induced VFS on *V*. *lantana* [18]. The site of Bleggio corresponds to the quadrate code 2602 in Gottardini et al. [19] (Table 1) and it is located at 824 m a.s.l., Lat 5,098,025 Long 1,604,029 (Gauss Boaga) (Fig 1). Based on the mean May-July AOT40 value estimated over the 4-year period from 2007 to 2010 in a previous study [19], Bleggio site resulted to be exposed to high ozone values (i.e. > 9,000 ppb h).

**Table 1:** Mean values (± s.d.) of leaf traits (LA: leaf area; DW: dry weight; SLA: specific leaf area; Chl_SPAD_: chlorophyll content measured with the chlorophyll meter SPAD; TrAd: adaxial trichomes density; TrAb: abaxial trichomes density; TrAd/TrAb: ratio between adaxial and abaxial trichomes density) sorted for site specific (aspect, shading) and plant (size, portion) variables. The p-value after one-way ANOVA analysis is reported.

	Aspect			Shading				Size			Portion			
	SE	NW	p	High	Mid	No	p	L	S	p	Basal	Mid	Apical	p
				n = 44	n = 69	n = 10		n = 55	n = 64		n = 14	n = 44	n = 61	
	n = 77	n = 42												
LA, cm^2^	29.7±8.10	31.1±12.65	ns	31.8±12.64	28.3±7.11	37.1±11.07	p<0.05	29.4±7.79	30.9±11.45	ns	27.4±9.88	27.1±6.83	33.1±11.02	p<0.01
DW, g	0.23±0.11	0.23±0.15	ns	0.23±0.15	0.22±0.09	0.34±0.18	p<0.05	0.25±0.11	0.22±0.13	ns	0.13±0.09	0.18±0.09	0.29±0.13	p<0.001
SLA, cm^2^ g^-1^	146.0±51.04	172.0±72.88	p<0.05	178.3±72.97	144.3±47.86	137.9±65.07	p<0.05	137.2±50.80	170.7±64.44	p<0.01	236.2±56.86	173.7±64.17	123.3±27.68	p<0.001
Chl_SPAD_, a.u.	42.6±8.78	38.5±3.56	p<0.01	39.1±4.76	42.7±8.90	38.8±4.36	p<0.05	43.8±9.02	38.9±5.24	p<0.001	34.2±3.46	41.2±7.49	42.7±7.58	p<0.001
TrAd (sqr), mm^-2^	1.70±0.70	1.7±0.76	ns	1.6±0.62	1.7±0.78	2.0±0.48	ns	1.9±0.57	1.6±0.80	p<0.05	1.6±1.03	1.5±0.54	1.9±0.70	p<0.01
TrAb, mm^-2^	13.0±5.75	10.0±4.08	p<0.01	9.9±4.34	12.9±5.87	13.7±3.31	p<0.01	14.9±4.82	9.5±4.58	p<0.001	10.0±6.38	11.2±4.83	13.0±5.40	ns
TrAd/TrAb	0.25±0.14	0.35±0.22	p<0.01	0.31±0.19	0.27±0.19	0.30±0.08	ns	0.26±0.13	0.31±0.22	ns	0.31±0.21	0.22±0.11	0.32±0.21	p<0.05

In order to describe the mean ozone levels in Trentino between May and July (i.e. during the main vegetative season and in agreement with the Directive 2008/50/EC) in 2020 and in the previous five years, hourly data of this pollutant measured by the six automatic monitors in the Trentino region were used and the AOT40 was calculated (data downloaded on February 2021 from: *https*:*//bollettino*.*appa*.*tn*.*it/aria/scarica*).

### 2.2 Experimental design

#### Plant selection and leaf collection

The selection of plants followed a two-step process and intended to favor pairwise comparisons between symptomatic/asymptomatic plants with the minimum possible noise that can be caused by differences in their location and micro-site characteristics. Initially, 30 *V*. *lantana* plants along the light-exposed edge of vegetation were surveyed and data were collected on: i) aspect of the light exposed part of the plant (in two classes: east and south, ES; west and north, WN); ii) plant shading (in three classes: 0; <50%; >50%); iii) plant height, as a proxy for plant age (in three classes: small <90 cm, medium 90–180 cm, large >180 cm; iv) ozone-induced VFS, in terms of frequency of symptomatic leaves per plant (in classes: 0; >0–5%; >5–50%; >50–100%). Second, a random selection of six symptomatic plants was operated, three each for the small and large plant height classes. The closest non-symptomatic plant of the same height class was selected for each symptomatic individual to allow a pairwise comparison. A random stem per each plant (n = 12) was collected, and all leaves were sampled per each stem portion (lower, medium and higher third, as a proxy for leaf age). All symptomatic leaves for each stem sector were included in the leaf trait measurement, and a balanced number of non-symptomatic leaves was randomly selected for the analysis. The field assessment on the n = 30 plants was carried out on 12 August 2020; leaf sampling and further measurements on the sub-set of n = 12 plants were carried out on 19 August 2020.

#### Leaf traits assessment

Each leaf was measured for a series of morpho-physiological traits. A Minolta SPAD 502 chlorophyll meter (Konica, Minolta) was used to measure the leaf SPAD values as a proxy of chlorophyll content (Chl_SPAD_). An RGB picture of each leaf on graph paper was analysed for Leaf Area (LA, cm^2^) estimation by ImageJ [20]. Subsequently, the amount of ozone-induced symptoms (in classes, 0: absent; 1: 1–5%; 2: 6–50%; 3: >50) was visually assessed as reported by Gottardini et al. [19]. Gel nail polish was used to create a leaf imprint of a fixed area on both the adaxial and abaxial surface of each leaf, at the three thirds of the lamina (six imprints per leaf; imprint area = 5.36 mm^2^). These imprints were then analysed for trichome density on both adaxial and abaxial surface via light microscope (DM2005, Leica Microsystems, Wetzlar, Germany) and normalized per mm^2^. Dry weight (DW, g) was obtained for each leaf after drying at 65°C to a constant weight (for 72 h) and Specific Leaf Area (SLA = LA/DW) was calculated.

### 2.3 Data analysis

Data analysis was performed with Rstudio (RStudio Team (2020), http://www.rstudio.com/ [cited 2021 December 2]) using the aov function, while figures were created via ggplot2 package. The assessment of data normality and heteroscedasticity was performed by visually assessing normal histograms of distributions and residuals vs fitted values. The density of trichomes in the adaxial surface showed skewness, thus square root transformation was applied to the dataset. Data were then subjected to analysis of variance (ANOVA) either one-way or factorial, depending on whether factor number and plant pairs (i.e., symptomatic and non-symptomatic) were taken into account as a block. Analyses were performed at leaf level. Principal component analysis (PCA) was carried out with the “ggbiplot” package. Fisher’s LSD post-hoc test was used for multiple comparisons. Nonparametric Mann–Whitney U test was performed for comparing symptomatic vs non-symptomatic leaves in symptomatic plants.

## 3. Results

### 3.1 Environmental data, ozone levels and experimental plant material

**Fig 2:**
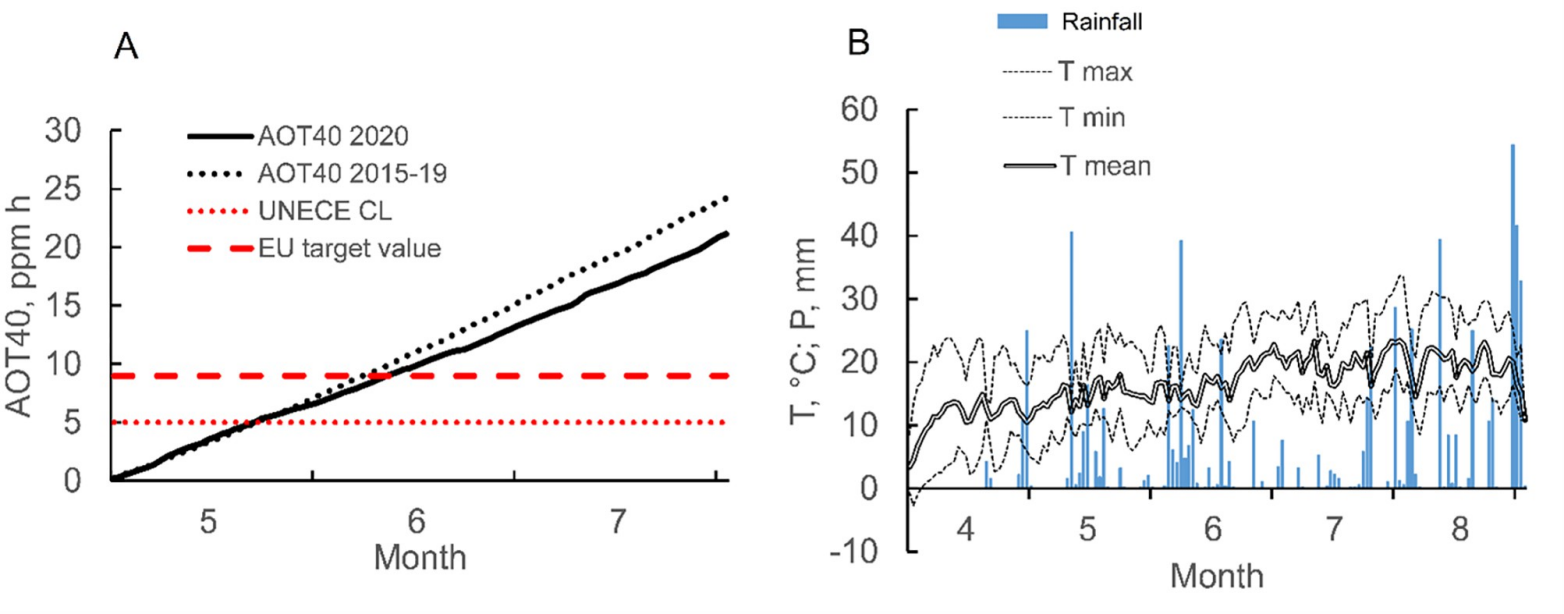
A) Time course of mean ozone exposure (AOT40, accumulated ozone over a threshold of 40 ppm h) measured by the six automatic monitors on the Trentino region in 2020 and in the previous 5 years (2015–2019), during the period 1 May—31 July. The red lines represents the target value for the protection of vegetation set by the Directive 2008/50/EC (dashed line) and the UNECE CL for forests (dotted line).). B) Average daily temperature (T mean), minimum and maximum temperature (T min and T max, respectively) as well as daily rainfall for the period 1 April—31 August measured in Tione (6 km far from Bleggio study site).

Mean May-July ozone concentration, measured by the six automatic monitors in the Trentino region in 2020, was 40.1 ppb, slightly lower than the previous 2015–2019 period (42.5 ppb). For the same May-July period, AOT40 resulted in 21.16 ppm h in 2020 and 24.20 ppm h as average over the 2015–2019 period (Fig 2A). The UNECE Critical Level for forests (5.00 ppm h) and the target value for the protection of vegetation defined by the Directive 2008/50/EC (9.00 ppm h) resulted to be exceeded at the end of May and mid of June respectively. Mean air temperature increased from an average of 15°C in April-June to an average 20°C in July-August (Fig 2B). Average daily precipitation was 4.3mm between April and August although a significant reduction in rainfall was observed for the period early-mid June to late July (1.77mm on average) (Fig 2B).

**Fig 3:**
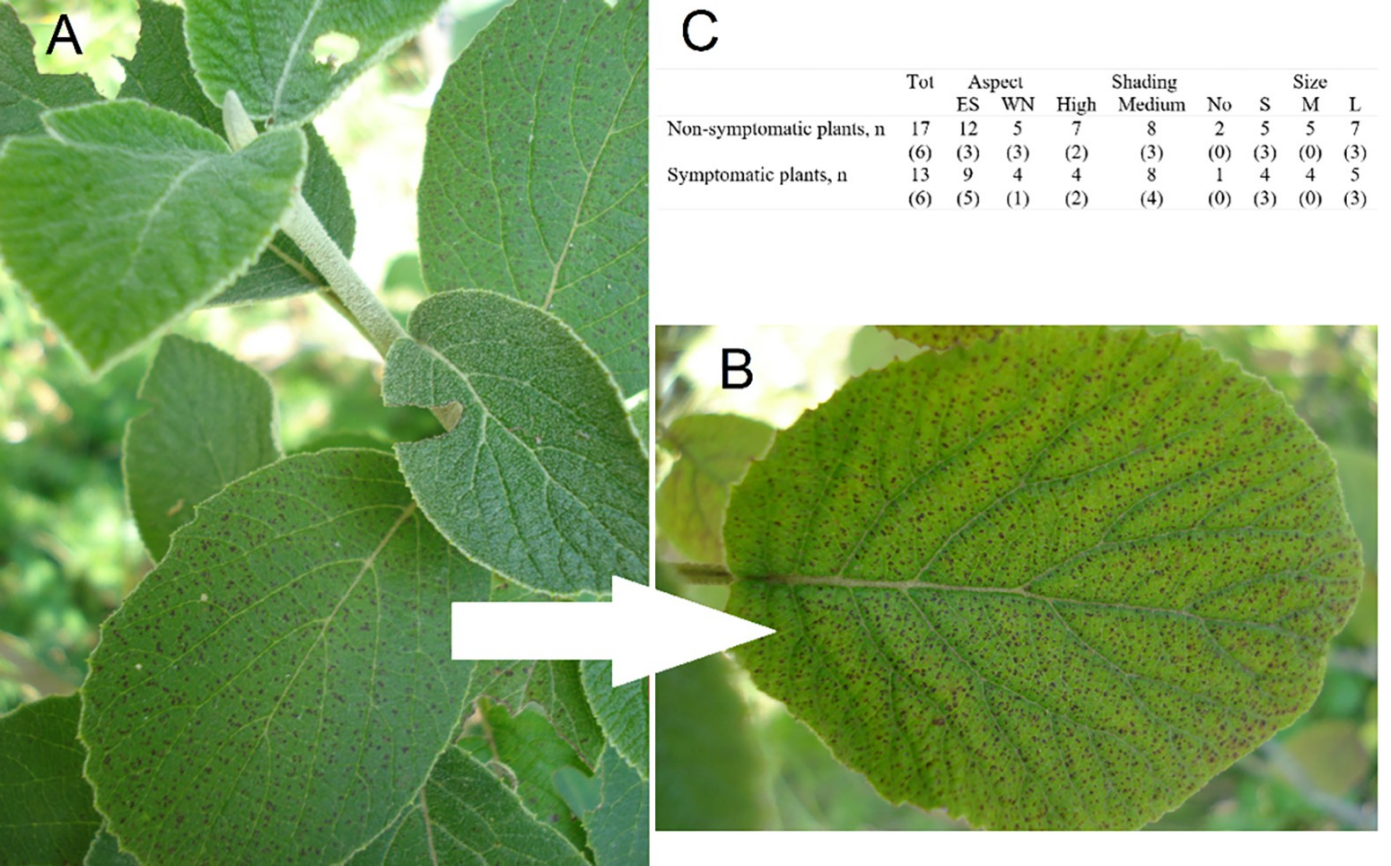
Example of VFS observed in *V*. *lantana* (A and B). In C, number of non-symptomatic and symptomatic *V*. *lantana* plants assessed at the Bleggio site, sorted for aspect, shading and size classes; the correspondent number of the subset plants, selected for the leaf trait investigation, is reported in brackets.

Overall, in the 2020 field assessment at Bleggio site on n = 30 plants, 43.3% of individuals showed VFS (Fig 3). Among the symptomatic plants, 69% and 31% presented low (1–5%) and medium (6–50%) frequency of symptomatic leaves, respectively; no plant showed a high frequency of symptomatic leaves. As for aspect, 43% of ES exposed, and 44% of WN exposed plants were symptomatic. Plants in full sunlight (no shading) showed symptoms on the 33% of individuals; in medium (i.e. shading < 50%) and high shading (i.e. shading > 50%), 50% and 36% of plants were symptomatic, respectively. No differences were observed in the frequency of symptomatic plants among plant sizes: 44% of both small and medium, and 42% of large plants were symptomatic (Fig 3). Among the twelve individuals selected for leaf trait investigations, eight were ES exposed (5 of which resulted symptomatic), and four were WN exposed (one of which was symptomatic); seven plants were in medium and four in high shading, respectively four and two of which were symptomatic (Fig 3).

### 3.2 Leaf traits, site-specific and plant-related characteristics

Table 1 reports the mean values of each leaf trait sorted for environmental and plant-related variables. Aspect had a significant effect on SLA, Chl_SPAD_, TrAb and TrAd/Ab ratio. In ES exposed plants, SLA resulted significantly lower than in WN exposed plants (p<0.05), while chlorophyll content and abaxial trichome density were higher (p<0.01). Shading had a significant impact on all the traits apart from TrAd and TrAd/Ab. In particular, a significant reduction in SLA and a significant increase in TrAb were observed in plants under high light exposure. Plant size was not significant for LA, DW and TrAd/Ab, while a significant effect was observed for the other traits. SLA values were lower for the tall plants than for the small ones, while Chl_SPAD_ and both adaxial and abaxial trichome density were higher in the tall plants than in the small ones. Apart from TrAb, the stem portion was significant for all the leaf traits. Specifically, LA, DW, Chl content and TrAd showed increasing values from the bottom to the upper stem portion, while SLA showed deceasing values.

### 3.3 Leaf traits and ozone-induced visible foliar symptoms

Table 2 reports the mean values for the functional leaf traits calculated for the leaves of non-symptomatic plants, and for both, the non-symptomatic and symptomatic leaves of the symptomatic plants separately. No difference was observed for leaf area (LA), while in symptomatic plants dry weight of symptomatic leaves was significantly (p<0.01) higher than of non-symptomatic leaves. Consequently, SLA resulted significantly (p<0.001) lower for symptomatic leaves. The trichome density on the adaxial leaf surface did not show significant differences among the three leaf categories. On the contrary, the abaxial trichome density was significantly higher for symptomatic compared to non-symptomatic leaves from both plant categories. This result is also reflected by the adaxial/abaxial trichome ratio, significantly lower for the symptomatic leaves in comparison to the leaves of non-symptomatic plants.

**Table 2:** Mean values ± S.E. of leaf traits and one-way ANOVA results (F and P value). Letters represent the significant differences after post-hoc Fisher’s LSD test. LA: leaf area; DW: dry weight; SLA: specific leaf area; Chl_SPAD_: chlorophyll content measured with the chlorophyll meter SPAD; TrAd: adaxial trichomes density; TrAb: abaxial trichomes density; TrAd/TrAb: ratio between adaxial and abaxial trichomes density.

Leaf trait	Non-symptomatic plants, n = 6	Symptomatic plants, n = 6		F	P value
	Non-symptomatic leaves, n = 51	Non-symptomatic leaves, n = 43	Symptomatic leaves, n = 25		
LA, cm^2^	29.9±1.35	28.7±1.44	33.3±2.18	1.726	0.183
DW, g	0.25±0.138a	0.18±0.116b	0.27±0.105a	5.571	0.005**
SLA, cm^2^ g^-1^	140.4±7.47a	189.0±10.40b	127.2±5.29a	13.069	<0.001***
Chl_SPAD_, a.u.	42.0±0.96	40.3±0.94	40.8±2.16	0.619	0.540
TrAd, n mm^-2^	3.8±0.35	3.3±0.47	2.8±0.36	1.810	0.168
TrAb, n mm^-2^	11.4±0.64a	11.2±0.76a	14.5±1.37b	3.535	0.032*
TrAd/TrAb	0.33±0.025a	0.28±0.032ab	0.20±0.023b	4.828	0.010**

When all non-symptomatic leaves were grouped (i.e. even non-symptomatic leaves from symptomatic plants were included in the non-symptomatic group), the significant differences for SLA, abaxial trichome and adaxial/abaxial trichome density ratio were confirmed. Leaf area, Chl_SPAD_ and adaxial trichome density did not show any significant difference.

**Fig 4:**
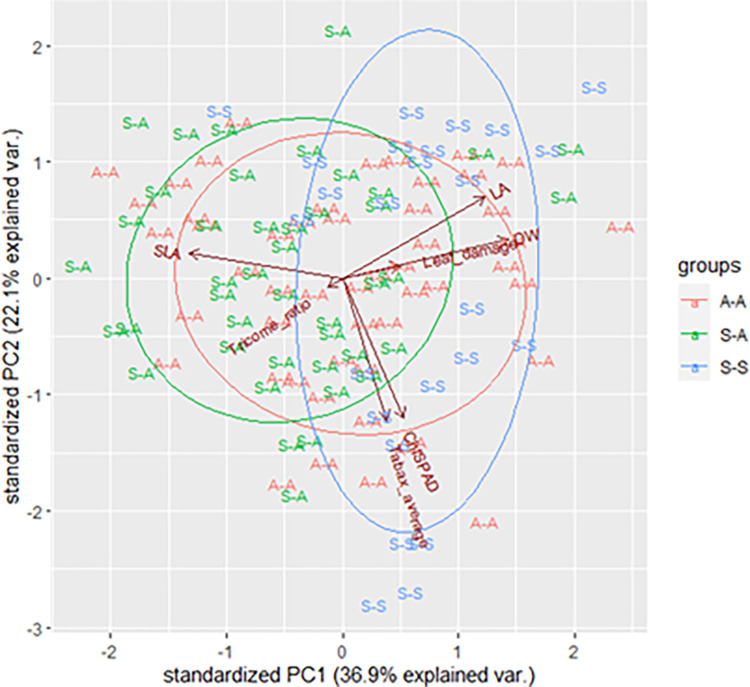
Principal component analysis (PCA) for the leaves of non-symptomatic plants (A-A), and for the non-symptomatic (S-A) and symptomatic leaves (S-S) of the symptomatic plants.

In the principal components analysis, PC1 and PC2 explained 36.9 and 22.1% of the variation (Fig 4), respectively, and total variance of 59%. PC1 loaded positively with LA, VFS, DW, TrAb and Chl_SPAD_, while PC2 loaded positively with SLA. Two main clusters with a significant overlap between non-symptomatic leaves of non-symptomatic plants (AA) and non-symptomatic leaves of symptomatic plants (SA) were observed, while a slightly separated cluster was present for symptomatic leaves of symptomatic plants (SS).

**Fig 5:**
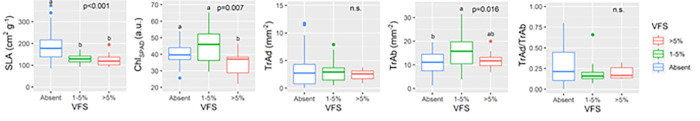
Leaf traits for symptomatic plants at different levels of visible foliar symptoms (VFS). SLA: specific leaf area; Chl_SPAD_: chlorophyll content measured with the chlorophyll meter SPAD; TrAb: abaxial trichomes density; TrAd: adaxial trichomes density; TrAd/TrAb: ratio between adaxial and abaxial trichomes density. Whiskers indicate the ranges of the minimum and maximum values; data were analysed with one-way ANOVA (n = 10–43) and p-value is shown in each graph. Different letters indicate significantly different values according to Fisher’s test.

In symptomatic plants, leaves with different levels of VFS severity expression were considered and significant differences between VFS levels were observed for SLA, Chl_SPAD_ and TrAb (Fig 5). Non-symptomatic leaves in symptomatic plants had higher (p<0.001) SLA than symptomatic leaves (both 1–5% and >5% VFS). Lower Chl_SPAD_ values were observed in leaves with >5% VFS (p = 0.007) when compared to non-symptomatic and slightly symptomatic (1–5%) leaves. An increase in trichome number was observed in leaves having a VFS of 1–5% and (although to a lower extent) in >5% VFS leaves (p = 0.016) and compared with non-symptomatic leaves. No significant differences were present for TrAd and trichome ratio.

**Fig 6:**
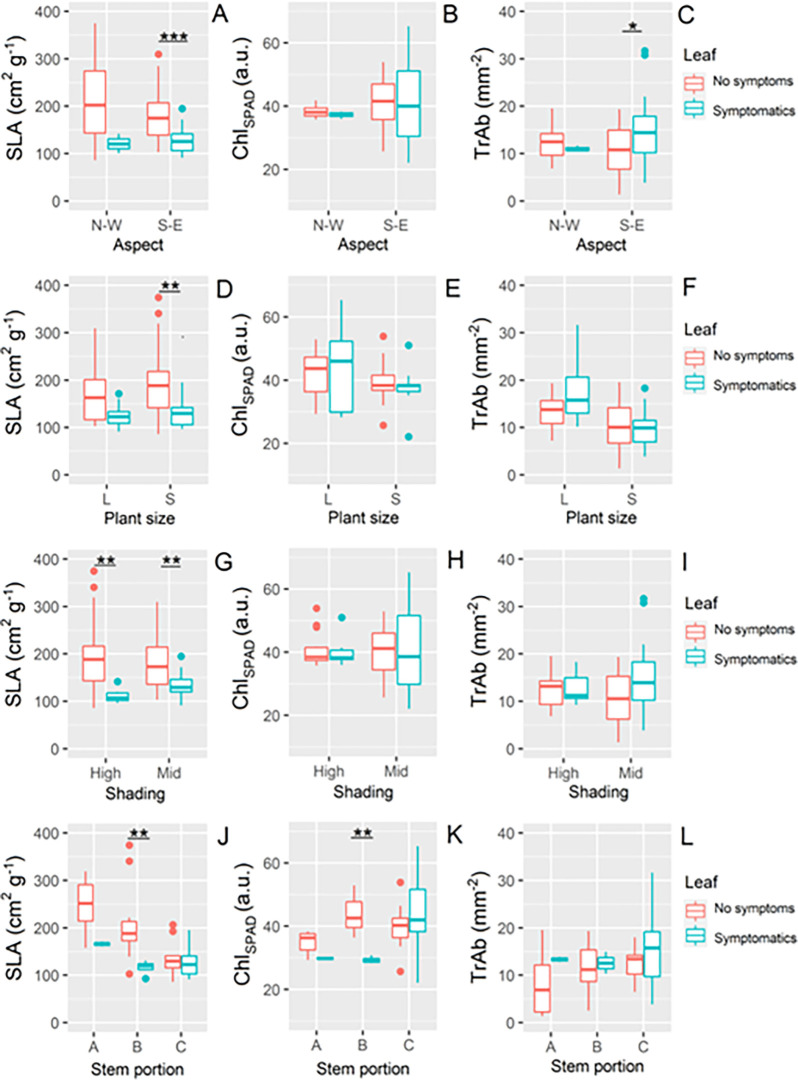
Comparison between the values of A) specific leaf area (SLA), B) chlorophyll content (Chl_SPAD_) and C) abaxial trichome density (TrAb) of symptomatic and non-symptomatic leaves of symptomatic plants, sorted for aspect, shading and stem portion categories. The results of Mann-Whitney U test are reported: *** p<0.001, ** p<0.01, *<0.05.

When only symptomatic plants were taken into account, significant differences were observed between symptomatic and non-symptomatic leaves and for different environmental and plant related factors (Fig 6). On ES exposed plants, symptomatic leaves were thicker (i.e., had a lower SLA) than non-symptomatic ones (p<0.001) accompanied by a higher trichome density on the abaxial surface (p<0.05). No significant difference was observed between symptomatic and non-symptomatic leaves in WN exposed plants, even if the symptomatic leaves were thicker than the non-symptomatic ones also at this exposure. Additionally, lower SLA values were observed in symptomatic leaves of both large (n.s.) and small (p<0.01) plants compared to non-symptomatic leaves, while either in high or mid-level of shading non-symptomatic leaves showed higher SLA (p<0.01) compared to symptomatic leaves. As for the stem portion, symptomatic leaves showed lower values in comparison to non-symptomatic ones in all the three heights, with a significant difference in the mid stem portion where also Chl_SPAD_ values of symptomatic leaves were lower when compared to non-symptomatic leaves (p<0.01).

## 4. Discussion

This study on the occurrence of ozone-induced VFS in *V*. *lantana* was carried out at the plot scale in order to minimize the differences of possible confounding factors as ozone levels, solar radiation, air temperature, water availability and soil conditions, and to resemble the typical scale of field assessment.

Firstly, observations of n = 30 *V*. *lantana* plants evidenced that ozone-induced symptom occurrence was not related to the individual exposure to the light. Indeed, the frequency of symptomatic plants did not differ significantly between full and moderate light exposure. In addition, the plant size (assumed here as a proxy for age) resulted to be not relevant for symptom occurrence.

Secondly, the functional leaf trait measurements on the subset of n = 12 plants allowed to explore the role of site-specific variables on the leaf plasticity and to study more in deep the relations between functional leaf traits and the development of ozone-induced VFS.

### 4.1 Site-specific variables and plant morphology modulate *Viburnum lantana* leaf traits

Assessment of ozone-induced VFS has been often considered a challenge, owing to the fluctuating environmental conditions and potential multiple stressors that concurrently affect plants in natural environments [21]. In the field, plants can be exposed to drought, high levels of soil salinity and heavy metals, nutrient imbalance, light stress and extreme temperature conditions [22], some of which often occurring simultaneously and rapidly changing [23]. This suggests that site-specific variation is a critical component in determining plant responses to ozone exposure. For instance, stomatal opening is influenced by several environmental factors such as light, water availability and vapor pressure deficit, which in natural ecosystems can vary spatially, due to e.g. shading, and temporally, within days, hours or minutes [23]; this can affect leaf conductance and in turn photosynthetic CO_2_ assimilation, growth and ozone uptake capacity. In our study, and regardless of ozone-induced VFS appearance and severity, significant differences were observed for several leaf traits in *V*. *lantana* plants subjected to contrasting environmental variables. For instance, ES exposed plants had lower SLA, higher chlorophyll content and increased density of trichomes on the abaxial leaf surface when compared to WN exposed plants. Similar trends were observed between non-shaded and shaded plants confirming that plant adaptation via plasticity in leaf shape and structure allows them to optimize photosynthesis to the surrounding environment [24]. For instance, in *Arabidopsis thaliana* plants exposed to different light conditions, one layer of palisade tissue was produced under low light intensity, whereas under high light two complete layers of palisade tissue were present [25]. The increase in the number of trichomes in sun-exposed plants belonging to the Myrtaceae family was previously associated with a strategy to reduce oxidative stress and limit water loss through a thicker leaf boundary layer [26]. In our study, we hypothesize that potential soil moisture deficit as well as temperature and light stress conditions were already present at the end of June (as shown in Fig 2 for temperature and precipitation), thus leading to the increase of trichome density as defensive strategy. Overall, our data suggest that even in a relatively small plot area (about two ha), site-specific variability may be significant and should be taken into consideration for sampling procedures and characterization of leaf traits under field conditions.

### 4.2 Ozone-induced VFS in *Viburnum lantana* are associated with thicker leaves and higher frequency of trichomes with a significant within-plant x environment effect

The tropospheric ozone concentrations observed in Trentino for 2020 were in line with those causing VFS in *V*. *lantana* [19]. Indeed, in our study, the typical VFS were present in *V*. *lantana* and this was associated with several changes in leaf traits when compared to non-symptomatic leaves.

Feng et al. [6] provided evidence of lower sensitivity to ozone in species with greater leaf mass per area (LMA; reciprocal of SLA). This evidence was hypothesized either as the result of a dilution effect (i.e. lower ozone load per unit leaf mass) or of a cross-protection, as the evolutionary adaptations of greater LMA is likely to induce protection against abiotic stresses and in turn to elevated ozone concentration. Yet, single leaf response to tropospheric ozone has been poorly studied and potential within-plant plasticity may exist owing to the different environmental conditions at which leaves are exposed in the foliage. *V*. *lantana* is considered a species with a marked sensitivity to ozone damage [14], potentially associated with a greater SLA when compared to other species [27]. Our data show that within-plant variation in SLA and between leaves of symptomatic and non-symptomatic plants exist in *V*. *lantana*. Previous work by Poorter et al. [28] showed that O_3_ exposure had minimal effects on leaf thickness of several species although different responses were observed between monocotyledons and dicotyledons, with the latter showing a trend in increasing leaf thickness when subjected to 80 nmol mol^-1^ O_3_ levels. In our study, when analyses were carried out at leaf level, SLA resulted to be significantly higher in non-symptomatic leaves than in symptomatic ones, and this difference remained evident also within the different site- and plant-related categories, corroborating the hypothesis that reducing SLA is a defensive strategy implemented to reduce stress damages at leaf level [29]. The alteration in leaf structure is an important mode of acclimation in many species under high temperature and heat and light stress, with increase in leaf thickness often being associated with enhanced stress tolerance [30, 31]. We speculate that symptomatic leaves under low irradiance levels (e. g. shaded leaves) showed lower frequency of VFS following a combination of reduced SLA and potentially low stomatal aperture (following low light interception). On the contrary, leaves at saturating or near-saturating light intensities could have a higher stomatal conductance and therefore a higher potential ozone uptake, linked to VFS appearance. For instance, sensitivity to O_3_ is considered higher in species with an intrinsically high SLA, possibly because thinner leaves were associated with a higher stomatal conductance that can lead to a higher ozone uptake [32]. In general, our data provide evidence of a leaf-specific response to ozone via SLA plasticity is present in *V*. *lantana* and this should be taken in consideration (along with environmental factors and leaf position) when large-scale assessments are carried out.

Similarly, increased in trichome production has been often associated with reduced stress damage, including increasing ozone concentration [33]. Indeed, a large body of evidence showed that glandular trichomes are involved in protection against ozone and significant positive effects on leaf transpiration and temperature regulation have been extensively shown in several species [8]. Mainly, glandular trichomes are able to store and exude a large number of metabolites such as unsaturated semi‐volatile compounds, volatile terpenoids and acylated sugars that, when released, are able to extinguish ozone before it enters the leaf [8]. On the contrary, non-glandular trichomes do not seem to be involved in any defensive role under elevated ozone concentration, although they can form a thick layer on the leaf surface that works as a mechanical obstacle against e.g. pathogens. Feng et al. [6] did not find any correlation between non-glandular trichomes and stomatal uptake of ozone, speculating that the low reactivity with ozone of the wax layer that covers non-glandular trichomes does not provide defense under elevated ozone levels. In addition, non-glandular trichomes have been associated with high leaf wettability [34], and therefore stomatal conductance maintenance under high vapor pressure deficit (VPD) conditions. In our work, only non-glandular trichomes were observed and their density was higher in symptomatic leaves, with higher VFS, and in plants of *V*. *lantana* exposed to higher irradiance level. We hypothesized that the increased number of trichomes in the upper section of the plant could be a strategy to reduce e.g. heat stress damage and maintain stomatal conductance under high VPD conditions in *V*. *lantana*. This, however, may allow higher O_3_ uptake and therefore a higher occurrence of VFS in leaves where non-glandular trichomes were more frequent. This hypothesis is supported by the significant difference between symptomatic and non-symptomatic leaves of plants exposed at ES, with a higher number of trichomes associated with the presence of VFS. Kollmann and Grubb [35] showed that *V*. *lantana* sun-exposed leaves had higher stomatal density than shaded ones, potentially leading to a higher ozone uptake per unit of mass/area and suggesting that combinations of site-specific variables determine the leaf sensitivity to ozone.

#### Conclusions

This study provides new insights on ozone VFS in *V*. *lantana* under field conditions in relation to leaf traits, site-specific and plant-related variables. Referring to the three study objectives, (i) a significant site-specific effect was observed on several leaf traits in *V*. *lantana* assessed at a small-scale, irrespective of ozone symptoms; (ii) the presence of ozone-induced VFS was associated with specific leaf traits such as lower SLA and higher abaxial trichome density, suggesting that potential strategies adopted for increasing tolerance under multiple stressors may be incidentally detrimental for the plant in circumstances of concurring increased ozone uptake capacity; (iii) site-specific and plant-related characteristics had significant effects on the frequency of ozone VFS, with aspect, plant size (i.e., plant age) and stem portion (i.e., leaf age) strongly driving their occurrence at leaf level. To conclude, our study provides evidence of a complex relationship between ozone foliar symptoms and environmental variables even at the very plots scale and within the same species and plant, suggesting the opportunity to consider such complexity into monitoring programs focusing on the impact of tropospheric ozone on vegetation.

## Supporting information

S1 Dataset (XLSX)
